# The Social Attribution Task-Multiple Choice (SAT-MC): A Psychometric and Equivalence Study of an Alternate Form

**DOI:** 10.1155/2013/830825

**Published:** 2013-06-20

**Authors:** Jason K. Johannesen, Jessica B. Lurie, Joanna M. Fiszdon, Morris D. Bell

**Affiliations:** ^1^Department of Psychiatry, Yale University School of Medicine, 300 George Street, Suite 901, New Haven, CT 06511, USA; ^2^Psychology Service 116-B, VA Connecticut Health Care System, 950 Campbell Avenue, West Haven, CT 06516, USA

## Abstract

The Social Attribution Task-Multiple Choice (SAT-MC) uses a 64-second video of geometric shapes set in motion to portray themes of social relatedness and intentions. Considered a test of “Theory of Mind,” the SAT-MC assesses implicit social attribution formation while reducing verbal and basic cognitive demands required of other common measures. We present a comparability analysis of the SAT-MC and the new SAT-MC-II, an alternate form created for repeat testing, in a university sample (n = 92). Score distributions and patterns of association with external validation measures were nearly identical between the two forms, with convergent and discriminant validity supported by association with affect recognition ability and lack of association with basic visual reasoning. Internal consistency of the SAT-MC-II was superior (alpha = .81) to the SAT-MC (alpha = .56). Results support the use of SAT-MC and new SAT-MC-II as equivalent test forms. Demonstrating relatively higher association to social cognitive than basic cognitive abilities, the SAT-MC may provide enhanced sensitivity as an outcome measure of social cognitive intervention trials.

## 1. Introduction

Human social behavior involves a complex interchange between self-perception, knowledge of social expectations, interpretation of subtle social cues, and inferences about the mental state of others. The study of social information processing, broadly termed social cognition [1], has increasingly become a priority area in psychiatric research and assessment. The profound effects of social impairment in schizophrenia may in part be explained by difficulties interpreting the emotions and actions of others and regulating one's own behavior in response to a dynamic social world. There is now considerable evidence to suggest that laboratory-based assessment of social cognition improves prediction of real world functional outcomes beyond what is captured by standard neuropsychological assessment [2–5].

Growing appreciation for the complexity of social behavior has guided the development of assessment methods to target discrete domains of social information processing, of which emotion processing, social perception, social knowledge, theory of mind (ToM), and attributional bias are the most widely studied in schizophrenia [1]. Despite general consensus regarding this categorization of social cognitive domains, determination of functional associations distinctly related to social information processing may be obscured by a generalized neurocognitive deficit common to this population [6–8]. Generalized deficit may confound assessment of even the most fundamental social cognitive processes. For example, tests of affect recognition simply require the examinee to view still photos or video images and select an emotion label from a list that best matches the emotion portrayed. Although these procedures would not be expected to place significant demand on higher-order cognitive abilities, 34% of the variance in affect recognition performance in schizophrenia was explained by attention and memory performance in one study [9] and 40% of the variance by executive function in another [10]. These and other studies [6, 7] suggest that deficits in emotion recognition task performance observed in schizophrenia are not only due to difficulty processing the emotional content of test stimuli but also to basic cognitive deficits. 

Tests of more complex theory of mind “ToM” or “mentalizing” processes, referring to the ability to correctly intuit mental and emotional states of others [11], typically present the examinees with vignettes of everyday social dilemmas to which they verbalize or write answers to questions about what they observed. Although this approach explicitly tests social information processing, the demands placed on basic verbal and cognitive abilities that may also influence test performance are less obvious. The Hinting Task, a widely used theory of mind measure [12, 13], has been found to correlate with structured verbal memory, processing speed, executive function, and thought disorder ratings in schizophrenia [13]. Therefore, in addition to basic cognitive capacities needed to comprehend and evaluate test stimuli, performance may also be affected by the examinee's ability to clearly articulate verbal responses. 

Given evidence for its mediating role in the relationship between basic neurocognition and community functioning [14–17], social cognition has been recommended as a proximal treatment target for intervention research [18]. In pursuit of this aim, we suggest that the careful evaluation of interventions targeting social information processing in schizophrenia will require laboratory tests that minimize dependence on basic neurocognitive abilities. Because neurocognitive impairment is a stable and enduring feature of schizophrenia, tests with significant cognitive demand may lack sensitivity to changes expected of interventions that focus narrowly on social information processing. 

The Social Attribution Task (SAT) possesses several qualities suitable for further development as a clinical trial outcome instrument. First, the SAT is based on an animation of moving geometric shapes enacting a social drama, originally developed by Heider and Simmel [19] for experiments on perception and the attribution of causality. The animation is silent and therefore places no demand on basic auditory processing or verbal comprehension. Visual stimuli used in social cognitive tests generally include complex visual displays and human faces, which may enlist visuomotor scanning [20] and facial encoding [21, 22] processes that are impaired in schizophrenia and could confound ascertainment of higher-order social problem solving abilities. The visual stimuli of the SAT consist of four simple geometric images (a rectangle, a small triangle, a large triangle, and a circle) presented monochromatically against a white background. Therefore, a second advantage of the SAT is that visual processing demands are also minimized. Although no social information is immediately connected with these universally recognizable stimuli, nearly all participants in Heider and Simmels' [19] seminal study derived rich themes involving human motivations, intentions, and roles based only on spatiotemporal relationships observed in the shapes' movements. Interestingly, a decrement in the capacity to detect this social content, as observed in autism spectrum disorders, results in descriptions entailing only the most salient features of the stimuli without anthropomorphic representation of their human qualities [23]. Therefore, a third advantage of the SAT is that it provides an implicit measure of social information processing in which there is no social bias influencing, or cue for guessing, what a correct or “socially appropriate” response would be. Tests that explicitly present social problems and query for solutions have been criticized on the grounds that individuals with profound social impairment can still perform at normal level [23]. This discrepancy between test performance and observed social function may be increased by test instructions that direct the examinee to focus on social elements of the problem, thereby introducing compensatory skills that are not utilized in naturalistic settings. Importantly, the SAT elicits spontaneous social attributions that are generated without apparent clues to the social nature of the task. For these reasons, the SAT may arguably provide a more authentic assessment of social cognitive capacities associated with ToM, specifically, the ability to internalize, interpret, and apply prior knowledge of relationships to new social context. 

To our knowledge, the first clinical studies using the SAT were conducted by Klin [23, 24] examining social attribution deficits in children and adults with Asperger's syndrome and high functioning autism. Notably, impaired SAT performance in these clinical samples was found to be unrelated to age, verbal IQ, or metalinguistic ability. Klin subsequently developed a multiple-choice response score system for the SAT, helping to further reduce dependence on verbal expression while also eliminating rating error associated with open-response tests. Given these qualities, we have included the SAT multiple-choice (SAT-MC) in ongoing functional outcomes research in schizophrenia. In a previous study examining diagnostic efficiency and external validity [25], SAT-MC performance alone accurately classified 75% of a large sample of chronic schizophrenia outpatients and healthy community adults. Furthermore, while the SAT-MC correlated with affect recognition, theory of mind, and social problem solving in schizophrenia, performance was not significantly related to attention and vigilance, processing speed, or verbal and visual learning abilities. Nevertheless, tests of executive functioning did correlate significantly with the SAT-MC, producing coefficients in the same range as tests of social cognition. We interpret this pattern of results as support for the premise that the SAT-MC places minimal demand on basic cognitive ability but may involve higher-order, integrative, and cognitive abilities that apply to social as well as nonsocial problem solving.

 The current study aims to further develop the SAT-MC for use in social cognitive interventions research. Among social cognitive measures commonly used in schizophrenia, there remains a need for tests that can be administered repeatedly in pre-post assessment [1]. In response, we have created an alternate version of the SAT-MC, hereafter referred to as the SAT-MC-II. The SAT-MC-II was constructed using the same format and similar geometric stimuli as the original, but object motion was altered to portray a new social drama. The present study examined the test equivalence of the SAT-MC-II as compared to the original version. In consideration of possible ceiling effects, a common psychometric limitation of current social cognitive tests [1], our preliminary data collection was conducted in a university student sample in which higher than average performance would be expected. The SAT-MC and SAT-MC-II were administered to separate groups, and equivalence was assessed according to score distributional properties, scale internal consistency, and strength of association with social cognitive and basic neurocognitive external validation measures. To detect variability in performance associated with psychological and personality characteristics that may affect social function, self-report ratings of social anhedonia, schizotypy, and social schema were also collected.

## 2. Method

### 2.1. Participants

Ninety-two undergraduate students were recruited at a large public university in Connecticut. All participants provided informed consent for procedures approved by the University's IRB. Eligibility only required enrollment for courses in the current semester. Participants enrolled in Introductory Psychology courses received credit in partial fulfillment of course requirements. Sample demographics (Table 1) are representative of the undergraduate student body at this university, with an average age of 19, predominately White Non-Hispanic, and from middle-income households. Eighty-five percent of the sample identified English as their first language, and all participants reported being fluent in English. 

### 2.2. Measures

All tests were administered in a group format using a lecture hall equipped with audio and video projection technology. To accommodate group administration and the requirement to complete all data collection within a 1-hour session, some measures were adapted in length or format from their original versions as described below.

#### 2.2.1. Social Attribution Task-Multiple Choice (SAT-MC; Klin, Unpublished Test)

**Figure 1:**
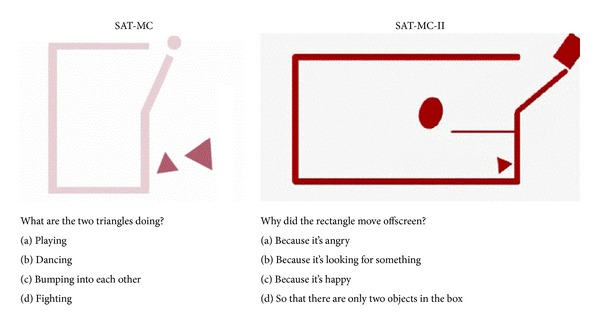
SAT-MC and SAT-MC-II sample items. Following presentation of the complete video, questions with multiple-choice response options are presented along with an image of the video section referred to by the question. Four response options are presented with each question with one describing the correct emotional intent, two describing action with incorrect emotional intent, and one describing object motion without emotional intent.

The Social Attribution Test stimulus is based on the work of Heider and Simmel [19] and later by Klin [23, 24], who subsequently adapted the multiple-choice (SAT-MC) response set as an alternative to the original open-response format. The SAT-MC consists of a 64-second animation of a social drama enacted by a large triangle, small triangle, and a small circle (see original on www.youtube.com under “Heider and Simmel Movie”). The animation is shown twice through in its entirety and is followed by short segments and multiple-choice questions about the actions depicted. Questions and answers are read aloud by a recorded voice embedded in the video, while the examinee reads along on the response form. In total, 19 questions are asked with 4 possible responses to each, one describing action with correct emotional intent, two describing action with incorrect emotional intent, and one describing object motion without emotional intent. A score of “11” was previously established as a cut-point for impairment based on distributions observed in schizophrenia (*M* = 11, SD = 4) and healthy (*M* = 15, SD = 3) community adults [25]. An alternate form, the SAT-MC-II, was created in our laboratory using the same timing and similar geometric figures as the original, but object motion was altered to create new social content. SAT-MC administration time was approximately 14 minutes, including a 20-second per-item time limit for response. Sample items from both versions are presented in Figure 1. 

#### 2.2.2. Psychosocial Functioning

The Revised Social Anhedonia Scale (RSAS; [26]) is a 40-item, true-false, self-report scale designed to assess deficits in the ability to experience pleasure from social interaction (e.g.: “Having close friends is not as important as many people say”). A 15-item short form was created for this study using the 10 most commonly endorsed (nos. 10,13,18,22,23,27,30,35,37,40) and 5 least commonly endorsed (nos. 1,2,15,19,32) items from the normative sample in which the original scale was developed. This revision was intended to optimize time efficiency, sensitivity, and discriminability in a healthy community sample. 

The Schizotypal Personality Questionnaire (SPQ; [27]) is a 74-item, true-false, self-report scale based on 9 factors representing the DSM-III-R symptoms of schizotypal personality disorder: ideas of reference, excessive social anxiety, odd beliefs, unusual perceptual experiences, odd-eccentric behavior, lack of close friends, odd speech, constricted affect, and suspiciousness. Raine [27] reported high internal consistency, test-retest reliability, and acceptable convergent, discriminant, and criterion validity in the original form. A 22-item short form (SPQ-B) was recently validated in nonclinical adolescents [28]. To optimize time efficiency and sensitivity to social function, the SPQ-B version was administered in this study with factors pertaining to social function (excessive social anxiety, no close friends, and constricted affect) supplemented by items from the original full-scale form. In total, this revised scale consisted of 40 SPQ items. 

The Bell Relationship Inventory for Adolescencets (BRIA; [29]) is a 50-item self-report assessment of interpersonal functioning in five domains: alienation, insecure attachment, egocentricity, social incompetence, and positive attachment. The BRIA has been standardized on adolescents aged 11 to 19 and demonstrates sensitivity to differences in object relations and emotional bonding between psychiatric and nonclinical samples. The egocentricity subscale, a measure of social schema, was previously found to be the most sensitive to SAT-MC performance using the adult version, the Bell Object Relations Reality Testing Inventory [25]. Current analysis of BRIA data was based on the egocentricity subscale. 

In addition to these self-report questionnaires addressing psychosocial function, participants were asked to report their involvement in university social life using a list of 16 organized activities available at this campus (e.g., club sports, student government, religious clubs, and sorority/fraternity). 

#### 2.2.3. Social Cognition

The Bell-Lysaker Emotion Recognition Test (BLERT; [30]) is an affect recognition task using short video vignettes of an actor reading three neutral scripts while portraying one of the seven emotions (happiness, sadness, anger, fear, surprise, disgust, and no emotion). Each script is crossed with each of the seven emotions, resulting in 21 video vignettes total and a possible score range of 0–21. For group administration, participants were provided with a response form on which they were asked to circle one of the seven emotion words in response to each item. Administration time was approximately 7 minutes with instructions. We have previously established a BLERT cutoff score of “≤16” as the impaired range [4] and reported a correlation between the BLERT and SAT-MC of *r* = 0.37 [25] in schizophrenia samples.

#### 2.2.4. Basic Cognition

The Picture Completion subtest of the Wechsler Adult Intelligence Scales, 3rd edition [31] entails 20 pictorial scenes in which one important element is missing. The examinee must use contextual information to identify the missing element. This test was included in the present study as a basic assessment of visual attention and problem solving that does not involve social information processing. The Picture Completion test was modified for group administration as follows. Participants were given the 20 picture stimuli on separate pages. The standard instructions were read aloud by the examiner but revised to instruct participants to draw a circle on each picture in the location where the missing element should be. The standard 20-second time limit was given for completion of each item. To pace timing for group administration, the picture stimuli were also projected on the lecture hall screen for 20 seconds each. Presentation and timing were automated using Microsoft PowerPoint software. The entire procedure took approximately 8 minutes to complete. Data were scored in raw score format due to modifications from the standard administration on which test norms are based. 

Estimated intellectual endowment was assessed based on Scholastic Aptitude Test (SAT) score. Participants self-reported their SAT score based on the following ranges: 1200–1440, 1441–1680, 1681–1920, 1921–2160, and 2161–2400. 

### 2.3. Statistical Analysis

Data were first inspected for distributional properties using the box plot function of SPSS with extreme outliers identified according to the default criteria (2 box lengths the interquartile range). No outliers were identified; therefore, all data were retained for statistical analysis. Groups completing the SAT-MC and SAT-MC-II were compared on demographic variables to determine sample equivalence using independent sample *t*-tests for continuous variables and chi-square for categorical variables. Independent samples *t*-test was used to compare mean SAT-MC test performance between groups. Scale internal consistency was assessed by coefficient alpha for full scale and by Spearman-Brown coefficients using the split-half method. Cross validation of the SAT-MC and SAT-MC-II was conducted by a comparison of the strength of Pearson correlations with external validation measures. Fisher's *r* to *z* transform was used to test for statistically significant differences in the strength of interrelationships between independent samples. A final analysis combined the samples to compare the association of SAT-MC and BLERT scores with demographic and validation measures using Williams's T2 statistic for dependent correlations [32].

## 3. Results

### 3.1. Distributional Properties

**Figure 2:**
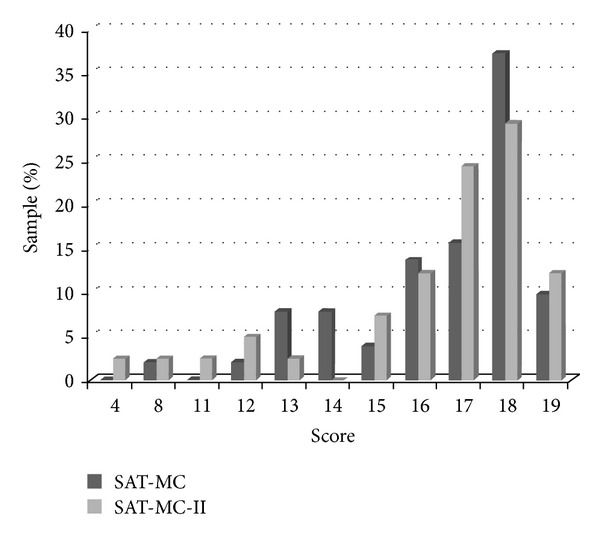
SAT-MC and SAT-MC-II raw score distributions. Score distributions are presented based on the frequency of each score in proportion to sample size.

There was no statistically significant difference on any demographic variable between participants completing the SAT-MC and SAT-MC-II (Table 1). SAT-MC and SAT-MC-II score distributions were essentially equivalent (Table 2) and did not differ statistically between samples; *t*(90) = .52, *P* = .61. On average, participants answered 16 of 19 items correctly. Five participants in each group performed at ceiling, and 1 SAT-MC versus 3 SAT-MC-II participants scored in the impaired range according to a previously established cut score [25]. Inspection of normality revealed that score distributions were negatively skewed in both versions (Figure 2). Accordingly, Blom transformation was applied to normalize this data prior to correlation analyses (Table 2).

Correlations between SAT-MC versions and demographic variables were explored in each sample (Table 3). No significant relationships were observed with age, intellectual endowment (SAT score), gender, or parental education.

### 3.2. Scale Reliability

The SAT-MC and SAT-MC-II differed in internal consistency, with alpha = 0.56 and 0.81, respectively. Split-half reliability coefficients were comparable to full-scale coefficients at 0.56 for SAT-MC and 0.83 for SAT-MC-II. For both versions, item analysis indicated that these reliability coefficients would not be substantially improved by removal of items.

### 3.3. Psychosocial Function

 SAT-MC and SAT-MC-II samples did not differ significantly with respect to SAS, SPQ, or BRIA scores. No significant associations were observed between SAT-MC-II performance and SAS or SPQ total self-report ratings (Table 4). Correlations with SPQ scales pertaining specifically to social function were also explored but yielded no significant relationships. A correlation between BRIA egocentricity scale ratings and the original SAT-MC was observed at trend level, but the strength of this association did not differ significantly from the nonsignificant correlation with the SAT-MC-II. 

The number of campus social activities reported ranged from zero to five, with 9.8% of the total sample reporting 0 activities, 25% reporting 1 activity, 25% reporting 2 activities, 23.9% reporting 3 activities, 10.9% reporting 4 activities, and 5.4% reporting 5 activities. The number of activities did not differ between samples and did not relate significantly to SAT-MC or SAT-MC-II performance (Table 4). 

### 3.4. Social and Basic Cognition

 Consistent with prior results obtained in a clinical sample [25], both versions of the SAT-MC correlated significantly with BLERT performance in this university sample (Table 4). The SAT-MC-II yielded a relatively, but not significantly, higher strength of association with the BLERT than the original SAT-MC. BLERT score distributions were essentially identical between the SAT-MC (*M* = 17.43, SD = 2.24) and SAT-MC-II (*M* = 17.63, SD = 2.50) samples, with generally high performance in both groups. 

To test discriminant validity with respect to basic cognitive function, correlations between both versions of SAT-MC and Picture Completion were examined. Small, nonsignificant, correlations indicate that SAT-MC and SAT-MC-II performance was not dependent on basic visual attention and problem solving abilities enlisted by this task (Table 4). Picture Completion performance was also nearly identical between participants completing the SAT-MC (*M* = 15.22, SD = 2.19) and SAT-MC-II (*M* = 15.38, SD = 2.28). 

### 3.5. SAT-MC and BLERT Comparison

Based on comparability observed between the SAT-MC and SAT-MC-II on sample test performance and relationships to external measures, the two test versions were considered essentially equivalent. To increase statistical power for detecting significant associations, correlational analyses were repeated in the combined sample collapsed by SAT-MC form. The correlation between SAT-MC and BLERT remained significant (*r*(92) = .34, *P* < .001), and no change was observed in relationship to other variables of interest (Table 5). A final analysis explored differences in the strength of association of the SAT-MC (combined sample) and BLERT across demographic and validation measures. The BLERT showed a pattern of small, nonsignificant correlations across demographic and psychosocial variables similar to the SAT-MC. However, a significant difference was observed between measures in relation to Picture Completion, in which case the BLERT showed higher association with this measure of basic cognitive function. 

## 4. Discussion

This study presents the first data collected in a university sample using the Social Attribution Test-Multiple Choice (SAT-MC) and a new test form, the SAT-MC-II. The SAT-MC and SAT-MC-II were compared for equivalence based on obtained score distributions in independent samples, on scale reliability, and on the pattern of interrelationships with demographic, personality, and cognitive characteristics of these samples. Across these levels of analysis, the SAT-MC-II performed comparably to the original test version, providing preliminary support for its equivalence as an alternate test form. Importantly, less than 11% of this university sample attained perfect performance across the two SAT-MC forms, suggesting adequate range and low likelihood of ceiling effects in clinical research applications. 

Scale reliability and split-half reliability for the SAT-MC were previously reported at 0.83 and 0.75, respectively, in a sample of schizophrenia patients and community healthy adults [25]. SAT-MC reliability in the current sample was relatively lower, with alpha = 0.56, whereas the SAT-MC-II (alpha = 0.81) was comparable to our previous data collection using the original SAT-MC. Lower internal consistency of the SAT-MC in these data likely reflects the restricted range of obtained scores (Table 2) and lower variance across items as compared to the SAT-MC-II distribution. 

Correlations with demographic variables indicated no association of either SAT-MC version to age, gender, or estimated intellectual status based on scholastic aptitude test (SAT) score. Although a narrow age range was studied in this sample, and intelligence would be expected to be high relative to the general population, these results are consistent with our prior research using the original SAT-MC in more diverse, older, and less educated community and clinical samples [25]. Socioeconomic status (SES) based on parental education was also assessed given the associations between SES and early cognitive and intellectual development [33, 34], but no significant relationships were found with the exception of a trend for a negative relationship between the original SAT-MC and father's education. We have no basis for interpreting this unanticipated finding but, for the purpose of evaluating scale equivalence, note that this correlation did not differ significantly from that obtained using the SAT-MC-II.

Variability in performance associated with social anhedonia, schizotypy, and social schema was examined. Of these variables, a single trend-level correlation was observed between the BRIA egocentricity scale and the original SAT-MC. This association is consistent with previous findings based on the egocentricity scale of the Bell Object Relations Reality Testing Inventory, the adult version of the BRIA, in an older sample [25]. This study is the first to investigate relationships between the SAT-MC and measures of schizotypy, thought to represent subclinical expressions of schizophrenia symptomatology. Whereas correlations with self-rated schizotypy and social anhedonia must be interpreted cautiously in these data, given that scales were modified and no effort was made to sample individuals with extreme scores reflecting the full range of the possible score distribution, these results are in line with prior findings suggesting a lack of association between the SAT-MC and symptoms in a chronic schizophrenia sample [25]. 

SAT-MC performance also showed no clear association to a global measure of participation in college activities. To more thoroughly evaluate the utility of this metric of social participation, we explored correlations with other psychosocial variables in the collapsed sample data. Activity number was found to correlate negatively with SPQ ratings of excessive social anxiety (*r*(92) = −.182, *P* < .05), no close friends (*r*(92) = −.203, *P* < .05), and with BRIA ratings of alienation (*r*(90) = −.195, *P* < .05). Therefore, despite lack of association with SAT-MC performance in this sample and only modest association with these other variables, less involvement in campus life did appear to reflect poorer social integration generally. 

 The main findings of the current analysis pertain to relationships of the SAT-MC and SAT-MC-II to independent measures of social and basic neurocognition. First, consistent with previous findings in a clinical sample of chronic schizophrenia patients [25], the original SAT-MC correlated with affect recognition performance ascertained by the BLERT. Importantly, SAT-MC-II performance was equally, if not slightly more highly, associated with this measure. Second, neither the SAT-MC nor SAT-MC-II demonstrated an appreciable association to basic visual attention and problem solving abilities assessed by Picture Completion. While these basic cognitive capacities are likely integral to SAT-MC performance, this pattern of results suggests that variability in SAT-MC performance in this healthy sample was more dependent on social cognitive abilities utilized in other tasks requiring inferences about social information (i.e., determination of emotional state from observed affect). Third, although the SAT-MC and BLERT related similarly to demographic and psychosocial measures, these measures differed significantly in their pattern of association to the Picture Completion task. Previous evidence suggests that the SAT-MC [25], and the original test on which it is based [23, 24], are reasonably sensitive to other indicators of social cognitive function but less influenced by basic verbal and cognitive abilities. Current findings, although based on limited assessment of basic cognitive function, do further support the argument that the SAT-MC is less dependent on basic, non-social, cognitive processes found to be associated with other established measures of social cognition.

The methodological choices made in designing the current study warrant some explanation. The primary aim of the study was to determine the equivalence between two forms of the SAT-MC. This was done in two separate samples, due to the possibility that immediate viewing of one test version could affect the responses on the second occasion if presented within a single session. We also favored this approach in terms of sample size to time constraints. Using the university student research pool, a 1-hour limit was placed on data collection, and it was not feasible to repeat testing on a second occasion. Without these constraints, other methods could have been employed, including the presentation of both the SAT-MC and SAT-MC-II to the same participants on separate testing occasions. However, we argue that the use of separate samples may still be the more appropriate approach for an initial data collection, given that unanticipated threats to internal validity resulting from carryover effects of one test version to the other are avoided. Moreover, the use of separate samples is a more conservative approach of testing equivalence, given the possibility that differences in sample characteristics between the two groups could affect SAT-MC scores as well as their relationships to other measures obtained. 

There are several limitations to this study. First, the sample was restricted in many ways, including age, education, and SES, and may not be representative of the general population in terms of intellectual and social competence. Therefore, lack of association between the SAT-MC and demographic characteristics in this sample may be due to restricted range, and this pattern of associations may not generalize to broader community samples. Moreover, although much of the research conducted on the SPQ and SAS has used university students, a much larger sample is typically assessed from which extreme groups are pooled. This approach was not possible in the current study because high scores on these measures were generally not observed. Therefore, it would be premature to conclude that social cognitive processes assessed by the SAT-MC are not affected by schizotypy. We also recognize that both the SAS and SPQ were modified from their original, validated forms. Although no changes were made to the original items used, use of fewer items may have decreased the sensitivity of these measures. Finally, this study is also the first instance of group administration for many of the measures used, and therefore it is not possible to determine at this time whether the current data yields the same results as would be obtained through individual testing.

A recognized limitation of currently available tests of social cognition is the lack of alternate forms, which may preclude repeated administration in clinical trial research. The current study aimed to address this need by evaluating an alternate form of the SAT-MC, an implicit measure of social cognition that may have the distinct advantage of reducing the neurocognitive demands required of other social cognitive test formats. Our preliminary data support the psychometric equivalence of this alternate form, the SAT-MC-II, and provide further evidence for the incremental specificity of the SAT-MC to social cognitive and relative independence from basic cognitive processes. 

## Figures and Tables

**Table 1:** Sample demographics.

	SAT-MC (*n* = 51)	SAT-MC-II (*n* = 41)	Test	Significance
Age^1^	19.27 (1.41)	19.03 (1.42)	*t*(77) = .78	*P* = .44
SAT score^2^				
1200–1440	5.9%	7.7%		
1440–1680	19.6%	30.8%		
1680–1920	51%	30.8%	*χ* ^2^(4) = 5.03	*P* = .28
1920–2160	21.5%	23%		
2160–2400	2%	7.7%		
Gender				
Male	39.2%	31.7%	*χ* ^2^(1) = .56	*P* = .45
Female	60.8%	68.3%		
Academic year				
Freshman	41.2%	63.4%	*χ* ^2^(3) = 4.77	*P* = .19
Sophomore	43.1%	26.8%		
Junior	13.7%	7.3%		
Senior	2%	2.5%		
Ethnicity				
Caucasian	68.6%	63.4%		
African-American	9.8%	7.3%		
Hispanic	9.8%	9.8%	*χ* ^2^(4) = 1.37	*P* = .85
Asian	5.9%	12.2%		
Other	5.9%	7.3%		

Parent education (M/F)^3^	Mother/father	Mother/father	Mother/father	Mother/father

Less than high school	1/2	1/0	*χ* ^2^(7) = 3.31/*χ* ^2^(7) = 6.53	*P* = .85/*P* = .48
Some high school	9/13	9/8		
Some college	8/6	3/6		
2 yr college degree	9/6	6/2		
4 yr college degree	14/15	12/12		
Master's	8/5	8/8		
Professional	0/3	1/4		
Doctorate	2/0	1/1		

^1^Age reported by *n* = 40 SAT-MC and *n* = 39 SAT-MC-II.

^2^Scholastic Aptitude test score, reported by *n* = 51 SAT-MC and *n* = 39 SAT-MC-II.

^3^Father's education reported by *n* = 50 SAT-MC.

**Table 2:** SAT-MC/-II and combined sample score distributions.

Statistic	SAT-MC *n* = 51	SAT-MC-II *n* = 41	SAT-MC + SAT-MC-II Combined sample
Range	11	15	15
Minimum	8	4	4
Maximum	19	19	19
Mean	16.53	16.24	16.40
Standard deviation	2.23	3.07	2.63
Skew	−1.55	−2.32	−2.15
Kurtosis	3.00	6.22	6.04
Skew after transform^1^	−.19	−.02	−.21
Kurtosis after transform^1^	−.28	−.39	−.31

^1^Blom-transformed values.

**Table 3:** SAT-MC and demographics correlations.

	SAT-MC (*n* = 51)	SAT-MC-II (*n* = 41)	Fischer's *z* ^1^
Age^2^	−.05	.05	*z* = −.43
SAT score^3^	.09	.09	*z* = .00
Gender	−.03	.20	*z* = −1.07
Mother Education	−.18	.13	*z* = −1.44
Father Education^4^	−.24^+^	.12	*z* = −1.67

Values reflect Pearson *r* correlation coefficients with two-tailed tests.

Statistical significance: ^+^ *P* < .10.

^1^Difference in the strength of correlations between SAT-MC versions with each demographic variable was tested using Fisher's *r* to *z* transform with a criteria for significance of *z* = ±1.96 at *P* < .05 (two-tailed).

^2^Age reported by *n* = 40 SAT-MC and *n* = 39 SAT-MC-II.

^3^Scholastic Aptitude test score, reported by *n* = 51 SAT-MC and *n* = 39 SAT-MC-II.

^4^Father's education reported by *n* = 50 SAT-MC.

**Table 4:** SAT-MC and external validation measures correlations.

	SAT-MC (*n* = 51)	SAT-MC-II (*n* = 41)	Fisher's *z* ^1^
SAS	−.06	−.12	*z* = .27
SPQ	−.11	−.15	*z* = .19
BRIA Ego^2^	−.24^+^	.04	*z* = −1.29
Social activities	−.03	−.08	*z* = .23
BLERT	.28*	.40**	*z* = −.63
Picture comp	.12	−.10	*z* = 1.02

Values reflect Pearson *r* correlation coefficients with two-tailed tests.

Statistical significance: ^+^ *P* < .10, *P < .05, **P < .01.

^1^Difference in the strength of correlations between SAT-MC versions with each validation measure was tested using Fisher's *r* to *z* transform with a criteria for significance of *z* = ±1.96 at *P* < .05 (two-tailed).

^2^BRIA data was incomplete for 2 participants in the SAT-MC-II sample, and useable data was collected for *n* = 39 in this group.

**Table 5:** SAT-MC and BLERT correlation comparison.

	SAT-MC + SAT-MC-II Combined sample	BLERT	William's T2^1^
Gender	.07	.00	*t* = −.58
Age^2^	−.01	.03	*t* = .30
SAT^3^	.08	.16	*t* = .66
SAS	−.07	.15	*t* = .66
SPQ	−.12	−.09	*t* = .24
BRIA Ego^4^	−.10	.01	*t* = .90
Social activities	−.06	−.06	*t* = .00
Picture comp	.02	.28**	*t* = 2.23*

Values reflect Pearson *r* correlation coefficients with two-tailed tests.

Statistical significance: *P < .05, **P < .01.

^1^Differences in the strength of correlations between SAT-MC, collapsed across versions (*N* = 92), and the BLERT were tested across demographic and validation measures by Williams's T2 statistic using a two-tailed *t* distribution, df = 89 unless otherwise indicated.

^2^Age reported by *N* = 79, df = 76.

^3^Scholastic Aptitude test score, reported by *N* = 90, df = 87.

^4^BRIA data was incomplete for 2 participants, and useable data was collected for *N* = 90, df = 87.
